# Occurrence of Florfenicol and Linezolid Resistance and Emergence of *optrA* Gene in *Campylobacter coli* Isolates from Tunisian Avian Farms

**DOI:** 10.1155/2024/1694745

**Published:** 2024-08-05

**Authors:** Manel Gharbi, Rihab Tiss, Chadlia Hamdi, Safa Hamrouni, Abderrazak Maaroufi

**Affiliations:** Group of Bacteriology and Biotechnology Development Laboratory of Epidemiology and Veterinary Microbiology Institut Pasteur de Tunis University of Tunis El Manar (UTM), Tunis 1002, Tunisia

## Abstract

*Campylobacter* species, especially *C. coli* and *C. jejuni*, have been associated with a range of human gastrointestinal diseases. During the last two decades, due to the irrational use of antibiotics in poultry farms, high rates of antimicrobial resistance have been globally reported in *C. coli* and *C. jejuni* isolates. Recently, acquired linezolid-resistance mechanisms have been reported in *Campylobacter* spp. isolates, which is a cause of concern to human health. In this study, we performed a retrospective analysis of 139 *C. coli* isolates previously collected from broilers (*n* = 41), laying hens (*n* = 53), eggs (*n* = 4), and environment (*n* = 41) to detect acquired genes implicated in linezolid resistance. Isolates were tested for their susceptibility to antimicrobial agents using the Kirby–Bauer disk diffusion assay. Chloramphenicol- and linezolid-resistant isolates were subjected to PCR screening for the following genes: *fexA*, *fexB*, *floR*, RE-*cmeABC*, *cfrA*, and *optrA*. The genetic relatedness of eight multidrug-resistant isolates was determined by multilocus sequence typing (MLST). Among the 139 *C. coli* isolates, high rates of resistance (57.55%–100%) were detected toward nalidixic acid, ciprofloxacin, erythromycin, azithromycin, ampicillin, chloramphenicol, linezolid, and kanamycin. Among 135 chloramphenicol-resistant isolates, the *optrA*, *cfr*, *fexA floR*, RE-*cmeABC*, and *fexB* genes were detected in 124 (124/135, 91.85%), 108 (80%), 105 (77.7%), 64 (47.4%), 56 (41, 48%), and 27 (20%) isolates, respectively. In addition, the majority of isolates harbored more than one of these genes. The selected eight isolates belonged to the same sequence type ST13450, which is a new sequence type (ST), not belonging to ST828 and ST1150 complexes. In conclusion, the emergence of *optrA* gene in *Campylobacter* spp. isolates makes this genus an *optrA* reservoir and vector to other pathogens such as *Staphylococcus aureus* and *Enterococcus* spp., which is a cause of concern for human and animal health.

## 1. Introduction

*Campylobacter* species have been associated with a range of human gastrointestinal diseases called campylobacteriosis, which is one of the four leading causes of diarrheal diseases worldwide [1]. Furthermore, reports of occasionally lethal extra-gastrointestinal infections such as reactive arthritis, irritable bowel syndrome, Guillain–Barré syndrome (GBS), and Miller Fisher syndrome, have also been recorded [2, 3]. Among the thirty-two species, *C. jejuni* and *C. coli* are the most common species that cause campylobacteriosis [1]. The main sources of infection for humans are meat products, particularly fresh and frozen chicken meat, and water [4]. The main reservoirs of *Campylobacter* species are poultry, domestic animals, and wild animals [1, 4, 5]. The majority of *Campylobacter* infections are self-limiting and normally do not require antibiotic therapy; however, patients who are immunocompromised or have severe and prolonged symptoms benefit from proper antibiotic therapy. For diarrhea caused by *Campylobacter* infection, fluoroquinolones (ciprofloxacin), macrolides (azithromycin, erythromycin, and clarithromycin), and tetracyclines are the preferred treatments; however, worldwide high rates of resistance to those antibiotics have been reported in *C. coli* and *C. jejuni* from humans as well as food-producing animals, thereby threatening public health.

Mutations in target genes (23S rRNA (A2075G or A2074C/G) and ribosomal proteins L4 (*rplD* gene) and L22 (rplV gene)), the CmeABC efflux system, or 23S rRNA methyltransferase (encoded by *erm*(B) gene) have been mainly identified in macrolide-resistant *Campylobacter* isolates [6]. Quinolone/fluoroquinolone resistance is mainly encoded by chromosome mutations in the genes regulating the synthesis of the *Campylobacter* multidrug efflux pump, named CmeABC, and in the quinolone resistance-determining region of DNA gyrase and/or topoisomerase IV [5]. The CmeABC efflux pump is able to extrude fluoroquinolones, macrolides, phenicols, and tetracyclines. However, over the past few decades, transferable mechanisms known as plasmid-mediated quinolone resistance (PMQR) have emerged, particularly in *Enterobacteriaceae*. The transferable PMQR encodes several types of genes known as qnr (qnrA, qnrB, qnrC, qnrD, qnrS, and qnrVC), qepA, oqxAB, and aac(6′)-Ib-cr genes [7, 8].

Concerning tetracycline resistance, the tet(O) gene, encoding a ribosomal protection protein, is often found in tetracycline-resistant *Campylobacter* isolates. Interestingly, *tet* (O) gene has been also reported in tetracycline-susceptible *C. jejuni* and *C. coli* isolates [9]. In addition, it seems that CmeABC efflux pump plays an important accessory role in mediating resistance to tetracycline among *Campylobacter* isolates [10].

The irrational use of antimicrobial agents in poultry production has enhanced selection of several other antimicrobial resistance mechanisms in *Campylobacter*. Florfenicol is a broad-spectrum antibiotic that is widely used in animals for both growth promotion and therapeutic reasons, which was the cause of increased rates of florfenicol resistance in *Campylobacter* spp. of animal origin in several countries like China [11]. Previously, only two transferable resistance proteins were known to give low-level florfenicol resistance (MIC ≤16 mg/L) in *Campylobacter*: the resistance-enhancing efflux pump CmeABC (RE-CmeABC) and the 23S rRNA methyltransferase Cfr(C). However, recently, the emergence of florfenicol exporter gene fexA in *C. coli* and *C. jejuni* isolates has been reported in China [12, 13]. *Campylobacter'*s *fexA* gene was frequently linked to mobile genetic elements and multidrug resistance genomic islands (MDRGIs) containing *tet(O)-catA-fexA-tet(L)-optrA* genes, which led to the co-transfer of *fexA* and other critically relevant antibiotic resistance genes as well as an increase in the emergence of multidrug-resistant *Campylobacter* [13, 14]. Interestingly, exclusively in China, *fexA*-positive *C. coli* and *C. jejuni* strains have been reported in chickens, ducks, goose, pigs, pigeon meat, human, and environment, indicating high ability of *fexA* gene and/or *fex*-positive isolates to spread within and between various ecosystems [11, 14, 15]. The cfr gene, which encodes 23S rRNA methyltransferase, confers resistance to five antimicrobial classes, namely, phenicols, lincosamides, oxazolidinones, pleuromutilins, and streptogramin A, known as PhLOPS_A_ phenotype. Among the five known types of the cfr gene family (cfr, cfr(B), cfr(C), cfr(D), and cfr(E)) in various genera, the plasmid-mediated multidrug resistance gene cfr(C) gene has been reported in *C. coli* and *C. jejuni* isolates from porcine, chicken, and cattle origin [16–18].

More recently, the Gram-positive (*Enterococcus* spp., *Staphylococcus* spp., and *Streptococcus* spp.) oxazolidinone (linezolid and tedizolid) resistance gene optrA has also been identified in *C. jejuni* and *C. coli* isolates [19, 20]. The optrA gene codes for an ABC-F protein which confers resistance by ribosome protection to not only oxazolidinones but also to fluorinated and non-fluorinated phenicols [19].

In this study, we performed a retrospective analysis and described the emergence of the RE-CmeABC, *fexA*, and *optrA* genes in *C. coli* from poultry in Tunisia.

## 2. Materials and Methods

### 2.1. Bacterial Strains

From 23 poultry farms located in North East Tunisia, 139 *C. coli* isolates have been previously reported from broilers (*n* = 41; cloacal swabs), laying hens (*n* = 53; cloacal swabs), eggs (*n* = 4; eggshell smears), and environment (*n* = 41; rays, nest boxes, and soil) [21–25]. All farms use an intensive floor hen rearing system with bird numbers ranging from 2000 to 18,000 hens per house.

### 2.2. Antimicrobial Susceptibility Testing

Antimicrobial susceptibility testing was performed on all isolates using the disk diffusion method according to the recommendation of the European Committee on Antimicrobial Susceptibility Testing guidelines [26]. The following antimicrobial agents were used (Oxoid, Basingstoke, UK): ampicillin (AMP, 10 *μ*g), amoxicillin/clavulanic acid (AMC, 10/20 *μ*g), gentamicin (GEN, 10 *μ*g), streptomycin (SMN, 10 *μ*g), kanamycin (K, 30 *μ*g), nalidixic acid (NAL, 30 *μ*g), ciprofloxacin (CIP, 5 *μ*g), tetracycline (TET, 30 *μ*g), erythromycin (ERY, 15 *μ*g), azithromycin (AZM, 15 *μ*g), chloramphenicol (CHL, 30 *μ*g), and linezolid (LIN, 10 *μ*g). The isolates were defined as multidrug-resistant if they exhibited resistance to at least three antibiotics belonging to three or more different antimicrobial families [27].

### 2.3. Screening of Genes Encoding Antimicrobial Resistance and Virulence

The genomic DNA of collected isolates was extracted using the boiling method [22, 28]. Chloramphenicol- and linezolid-resistant isolates were subjected to PCR screening for the following genes (Table 1): *fexA* [11], *fexB* [30], *floR* [29], RE-*cmeABC* [12], *cfrA* [31], and *optrA* [32].

### 2.4. Genetic Relatedness of Isolates by Multilocus Sequence Typing (MLST)

Eight multidrug-resistant (MDR) isolates were selected to determine their clonal lineage by MLST. PCR amplicons identifying seven allele loci (aspA, glnA, gltA, glyA, pgm, tkt, and uncA) were obtained for each isolate by using the primers provided in PubMLST database (https://pubmlst.org/organisms/campylobacter-jejunicoli/primers). After sequencing of PCR products, ST profiles were assigned by submitting the sequences to the PubMLST database using the submission database.

## 3. Results and Discussion

Globally, the rate of antibiotic resistance in *Campylobacter* isolates has increased over time. Of particular concern is resistance to macrolides and fluoroquinolones, which are the cornerstones of treatment for human infections. In addition, *C. coli* and *C. jejuni* isolates have showed high ability to acquire multiple resistance genes encoding resistance particularly toward tetracyclines, aminoglycosides, and fluorinated and non-fluorinated phenicols. The continuing application of antibiotics in livestock, especially chicken, exacerbates this issue [33].

Although several molecular mechanisms of antimicrobial resistance are specific to *Campylobacter* genus, several studies have showed the acquisition of antimicrobial resistance genes from Gram-positive genus, especially from *Enterococcus* spp., *Staphylococcus* spp., and *Streptococcus* spp. [33].

**Figure 1:**
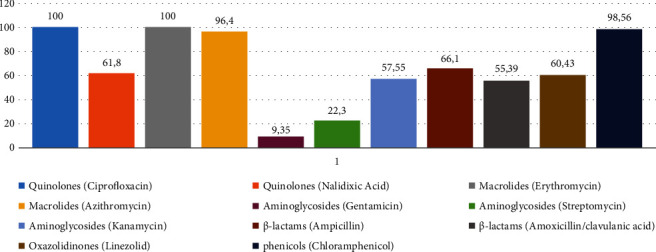
Frequencies of antimicrobial resistance detected in the 139 *C. coli* isolates.

Among the 139 *C. coli* isolates, high rates of resistance were detected toward nalidixic acid (*n* = 86, 61.8%), ciprofloxacin (*n* = 139, 100%), erythromycin (*n* = 139, 100%), azithromycin (*n* = 134, 96.4%), ampicillin (*n* = 92, 66.1%), chloramphenicol (*n* = 137, 98.56%), linezolid (*n* = 84, 60.43%), and kanamycin (*n* = 80, 57.55%) (Figure 1). These rates are similar or even higher to those reported in poultry, humans, and environment worldwide [34, 35]. However, the lowest resistance rates were observed for gentamicin and streptomycin (Figure 1) as previously reported [34, 35]. Taken together, according to the antimicrobial profiles of all isolates, the majority of isolates (>60%) showed MDR phenotypes. Worldwide, the MDR phenomenon represents another widespread and serious issue, which led to failure of even the most recent types of effective antibiotics. Within the *Campylobacter* genus, there have been numerous reports of multidrug resistance, regardless of the source of the isolates, especially toward ciprofloxacin, nalidixic acid, ampicillin, tetracycline, doxycycline, erythromycin, azithromycin, lincosamide, clindamycin, gentamicin, kanamycin, and streptomycin [36].

Ciprofloxacin resistance in *Campylobacter* arises quickly after exposure to antibiotics in the fluoroquinolone class, including enrofloxacin, which is unlawfully and excessively used in poultry production in Tunisia. Unfortunately, official statistics regarding the use of antibiotics by veterinarians or the amount of antibiotics sold for use in livestock are lacking in Tunisia, which makes it more difficult to draw a comprehensive knowledge on the potential relationship between the use of antibiotics and the emergence of antibiotic resistance in livestock production. The issue of fluoroquinolone resistance in *Campylobacter* poses a public health risk since fluoroquinolones, especially ciprofloxacin, are the preferred empirical treatment choices for campylobacteriosis in humans [28, 37]. Furthermore, a great deal of data points to a direct transfer of fluoroquinolone resistance from poultry to humans [38]. As a result, the use of this antibiotic in chicken production was banned in 2005 [39].

Treatment for human campylobacteriosis primarily consists of erythromycin and azithromycin. Consequently, the observed high frequency of resistance to erythromycin (100%) and to azithromycin (96.4%) in avian *C. coli* isolates is also a cause of concern to human health. Indeed, evident spreading of resistant isolates to humans through the food chain or environment has been reported [33].

The high resistance frequency toward chloramphenicol (*n* = 137, 98.56%) is somehow expected; in fact, despite its official restriction, farmers still use it in poultry production because of its affordable cost. In the European Union (EU), the use of chloramphenicol in veterinary medicine is currently restricted to pets and non-food-producing animals. The EU banned its use in animals raised for food in 1994. Protection of the consumer from probable negative effects resulting from chloramphenicol residues in food animal carcasses was the primary justification for this restriction. Chloramphenicol has been employed in human medicine over years; however, its fluorinated derivative florfenicol is one of the most frequently used antimicrobials in the treatment of animals (for respiratory and intestinal infections) and food animal production. Resistance to chloramphenicol is mediated by several mechanisms, where some of them mediate cross-resistance to florfenicol [40].

Interestingly, linezolid (belonging to oxazolidinone family) is the antibiotic of the last resort for treating clinical infections caused by MDR Gram-positive bacteria, including methicillin-resistant *Staphylococcus aureus* (MRSA), penicillin-resistant *Streptococcus pneumoniae*, and vancomycin-resistant *Enterococcus species* [41]. However, linezolid has not been approved for use in the livestock or poultry industries [41, 42]. Therefore, the high prevalence of linezolid resistance (60.43%) detected in this study would be associated to other factors rather than linezolid use [43].

Among the conserved 137 chloramphenicol-resistant isolates, 135 isolates were able to re-grow on selective medium and therefore were further studied by PCR. The *optrA*, *cfr*, *fexA floR*, RE-*cmeABC*, and *fexB* genes were detected in 124 (124/135, 91.85%), 108 (80%), 105 (77.7%), 64 (47.4%), 56 (41, 48%), and 27 (20%) isolates, respectively (Table 2). In addition, the majority of isolates harbored more than one of these genes.

The optrA gene codes resistance to linezolid, florfenicol, and chloramphenicol [13, 19]. In Tunisia, this gene has been previously reported in *Enterococcus* spp. isolates recovered from wastewater, chicken feces, retail chicken meat, and neutropenic patients [44–47]. As mentioned above, oxazolidinones are not used in livestock or poultry industries; therefore, the acquisition of the *optrA* gene by the *C. coli* isolates might not be directly linked to selective pressure by linezolid or other oxazolidinones [13]. Previous Tunisian studies have reported *optrA*-harboring enterococci colonizing the intestine of chickens; consequently, it is plausible that avian *C. coli* isolates are able to acquire the *optrA* gene from avian *optrA*-positive enterococci isolates by horizontal transfer of mobile genetic elements [45, 47]. Indeed, Frye et al. [48] have highlighted that *C. coli* and *Enterococcus* co-isolated from swine fecal samples had *tet*(O) and *aphA*-3 genes detected in common suggesting horizontal exchange of antimicrobial resistance genes between these bacteria or there may be a common source of those genes in the swine environment. This is also argued by the genetic studies on the genetic environments of *optrA* in *C. coli* and *C. jejuni* showing that multidrug resistance genomic islands (MDRGIs) containing *optrA* gene were most likely not indigenous to *Campylobacter* (GC content 31%) but derived from other bacteria with a higher GC content, such as *E. faecalis* and *E. faecium* (*E*. *faecalis* T5 (GenBank accession no. KB944666.1) and *E. faecium* ZY2 (GenBank accession no. CP039729.1) with 37.37% and 37.94%, respectively) [11, 19, 20]. The genetic studies showed also that the *optrA* regions found in the *C. coli* genomes closely resembled regions previously found on *E. faecalis* plasmids [13]. In addition, as OptrA encodes also resistance to florfenicol and chloramphenicol, the emergence of this mechanism of resistance to oxazolidinones and phenicols might be a logical response to the excessive use of phenicols in poultry production.

As an intestinal commensal of chickens, *Campylobacter* under selective pressure by phenicols has developed other molecular mechanisms to escape the bactericidal effect of these antibiotics. One of those mechanisms is the export of chloramphenicol or florfenicol from the bacterial cell mediated by either specific transporters and/or multidrug transporters. In *Campylobacter*, *fexA*, *floR*, and RE-*cmeABC* genes encoding those transporters have been increasingly reported [11, 14, 15, 19, 33]. However, to the best of our knowledge, this is the first report of *fexB* in *Campylobacter* [15, 19].

Others drivers of multi-antimicrobial resistance in *Campylobacter* are cfr and RE-*cmeABC* genes mediating resistance to phenicols-lincosamides-oxazolidinones-pleuromutilins-streptogramin A (PhLOPS_A_ phenotype) and phenicols-macrolides-fluoroquinolones-tetracyclines by multidrug efflux pump. Among the five types of the cfr gene family that have been reported: cfr, cfr(B), cfr(C), cfr(D), and cfr(E), only cfr(C) has been identified in *Campylobacter* [49]. The CFR(C) protein showed 55.1% or 54.9% identity to the original Cfr (GenBank accession no. CAC04525.1) from *Staphylococcus sciuri* and Cfr(B) (GenBank accession no. AKV84429.1) from *E. faecium*, respectively [50]. In our study, the *cfr* gene was found in 108 chloramphenicol-resistant isolates, which is to the best knowledge its first report in *Campylobacter*. Previous studies showed that RE-cmeABC was significantly linked with multidrug resistance among *C. jejuni* and *C. coli* [12, 51]. Although this resistant form of efflux pump has been identified globally in *C. jejuni* since at least 2014 [12], this study is one of the first reports of the RE-cmeABC form of the pump in *C. coli* in Tunisia.

It is worthy to note that the distribution of the investigated resistance genes, except *optrA* gene, was dependent to the origin of the isolates. The *fexA* and *cfr* genes were significantly more prevalent in isolates collected from layer hens, eggs, and environment than those isolates from broiler hens. However, *fexB* gene was more prevalent in isolates from broiler hens. Difference of gene distribution was also observed for RE-*cmeABC* gene which was more prevalent in isolates from broiler hens and environment. Similarly, the *fexB* was more prevalent in isolates from broiler hens than those from other origins. This finding may be related to the dissemination of particular *C. coli* clones or specific plasmids harboring those genes in each ecological niche.

In order to better understand the epidemiology of the phenicol-resistant isolates, eight isolates were selected (two from each origin) according to the occurrence of the majority of genes and their antimicrobial resistance profiles and their MLST was determined (Table 3). In Table 3, genes encoding other resistance markers (quinolones/fluoroquinolones, tetracyclines, CmeABC efflux system, and macrolides) and virulence factors were performed in our previous studies [21–25]. All isolates belonged to the same sequence type ST13450, which is a new ST. The singleton ST13450 is closely related to ST828 and ST1150 complexes, which account for the great majority of strains found in agriculture and human disease [52]. This finding is not surprising since it is well documented that *C. coli* from various origins showed a low genetic heterogeneity in contrast to *C. jejuni* [52, 53].

## 4. Conclusion

This study highlights the high ability of *C. coli* to acquire several genes encoding antimicrobial resistance especially to clinically relevant antibiotics. The distribution of those genes between isolates of various origins seems to be origin depending, which highlights possible circulation of specific *C. coli* clones or plasmids. Furthermore, it seems that heavy antibiotic use in avian farms drives the selection and the spread of MDR isolates. The emergence of *optrA* gene in *Campylobacter* spp. isolates makes this genus an *optrA* reservoir and vector to other pathogens such as *Staphylococcus aureus* and *Enterococcus* spp., which is a cause of concern for human and animal health.

## Figures and Tables

**Table 1:** Primers used for PCR reactions.

Gene	Primers (5′-3′)	Conditions of amplification	Size (bp)	References
*floR*	F-CGCCGTCATTCCTCACCTTC R-GATCACGGGCCACGCTGTGTC	94°C/5 min; 35 cycles: 94°C/30 s, 50°C/30 s, 72°C/1 min; 72°C/5 min	215	[29]
*fexA*	F-TTGGGAAGAATGGTTCAGGG R-ATCGGCTCAGTAGCATCACG	95°C/5 min; 30 cycles: 95°C/30 s, 50°C/30 s, 72°C/30 s; 72°C/5 min	977	[11]
*fexB*	F-ACTGGACAGGCAGGCTTAAT R-CCTGCCCCAAGATACATTGC	95°C/5 min; 30 cycles: 95°C/30 s,57°C/30 s, 72°C/30 s; 72°C/5 min	319	[30]
*cfr*	F-GGGAGGATTTAATAAATAATTTTGGAGAAACAG R-CTTATATGTTCATCGAGTATATTCATTACCTCATC	93°C/5 min; 35 cycles: 93°C/1 min, 58°C/1 min,72°C/1 min; 72°C/5 min	580	[31]
*optrA*	optrA-F-AGGTGGTCAGCGAACTAA optrA-R-ATCAACTGTTCCCATTCA	95°C/3 min; 30 cycles: 94°C/30 s, 55°C/30 s, 72°C/90 s; 72°C/5 min	1395	[32]
RE-*cmeABC*	F-CGTATTGCACGATTATTTGGAC R-ATCGTTATCAAACCCTCTATGTGCC	94°C/5 min; 35 cycles: 94°C/1 min, 54°C/1 min, 72°C/1 min; 72°C/5 min	742	[12]

**Table 2:** Occurrence of phenicol/linezolid-encoding genes in 135 chloramphenicol-resistant *C. coli* isolates.

Origin of resistant isolates (*n* of resistant isolates/N of isolates per sample)	Phenicol/linezolid-associated genes (*n*/% among total resistant isolates *per* sampling origin)					
	*floR* (*n* = 64) (%)	*fexA* (*n* = 105) (%)	*fexB* (*n* = 27) (%)	*cfr* (*n* = 108) (%)	*optrA* (*n* = 124) (%)	RE-*cmeABC* (*n* = 56) (%)
Broiler hens (38/41)	22/53.36	22/53.36	12/29.26	22/53.36	38/92.68	25/60.97
Layer hens (41/49)	32/65.30	41/100	9/18.36	41/100	41/83.67	19/38.77
Eggs (4/4)	0/0	4/100	0/0	4/100	4/100	3/75
Environment (41/41)	10/24.39	38/92.68	6/14.63	41/100	41/100	9/21.95

**Table 3:** Phenotypic and genotypic traits of eight multidrug-resistant *C. coli* isolates.

Isolate code	Year	Origin	Antimicrobial resistance profiles	Acquired antimicrobial resistance genes and mutations in GyrA and 23S rRNA genes	Virulence genes	ST
CC01	2015	Broiler hens	AM-AMC-NAL-CIP-ERI-TET- CHL-LIN	*floR, fexA*, *optrA*, *cfr*, RE-*cmeABC*, *tetO*, *bla*_OXA−61_, *aac(6′)-Ib-*cr, Thr-86-Ile, Ala-2074-Cys	*cadF*, *ciaB*, *racR*, *flaA*, *dnaJ*, *cdtA*, *cdtB*, *cdtC*, *virB11*, *pldA*, *cgtB*	13450
CC02	2015	Broiler hens	AM-AMC-NAL-CIP-ERI-TET- CHL-GEN	*floR, fexA*, *cfr*, RE-*cmeABC*, *tetO*, *bla*_OXA−61_, *aac(6′)-Ib*, Thr-86-Ile, Ala-2075-Gly	*cadF*, *ciaB*, *racR*, *flaA*, *dnaJ*, *cdtA*, *cdtB*, *cdtC*, *virB11*, *pldA*, *cgtB*	13450
CC03	2017	Layer hens	AM-AMC-NAL-CIP-ERI-TET-CHL-LIN	*floR, fexA*, *fexB*, *optrA*, RE-*cmeABC*, *tetO*, *bla*_OXA−61_, *ermB*, *aac(6′)-Ib-*cr, Thr-86-Ile, Ala-2074-Cys	*cadF*, *ciaB*, *racR*, *flaA*, *dnaJ*, *cdtA*, *cdtB*, *cdtC*, *virB11*, *pldA*, *ceuE*, *cgtB*	13450
CC04	2017	Layer hens	AM-NAL-CIP-ERI-TET-CHL- LIN	*floR, fexA*, *fexB*, *optrA*, RE-*cmeABC*, *tetO*, *bla*_OXA−61_, *aac(6′)-Ib-*cr, Thr-86-Ile, Ala-2074-Cys	*cadF*, *ciaB*, *racR*, *flaA*, *dnaJ*, *cdtA*, *cdtB*, *cdtC*, *virB11*, *pldA*,, *ceuE*, *cgtB*	13450
CC05	2017	Eggs	AM-NAL-CIP-ERI-TET-CHL- LIN	*floR, fexA*, *fexB*, *optrA*, RE-cmeABC, *tetO*, *bla*_OXA−61_, *aac*(6′)-Ib, Thr-86-Ile, Ala-2074-Cys	*cadF*, *ciaB*, *racR*, *flaA*, *dnaJ*, *cdtA*, *cdtB*, *cdtC*, *virB11*, *cgtB*	13450
CC06	2017	Eggs	AM-NAL-CIP-ERI-TET-CHL- LIN	*floR, fexA*, *fexB*, *optrA*, RE-*cmeABC*, *tetO*, *bla*_OXA−61_, *ermB*, *aac*(6′*)-Ib-*cr, Thr-86-Ile, Ala-2074-Cys	*cadF*, *ciaB*, *racR*, *flaA*, *dnaJ*, *cdtA*, *cdtB*, *cdtC*, *virB11, cgtB*	13450
CC07	2018	Environment	AM-AMC-NAL-CIP-ERI-TET- CHL-GEN-LIN	*fexA*, *cfr*, *optrA*, RE-*cmeABC*, *tetO*, *bla*_OXA−61_, *ermB*, *aac*(6′*)-Ib-*cr, Thr-86-Ile, Ala-2074-Cys	*cadF*, *ciaB*, *racR*, *flaA*, *dnaJ*, *cdtA*, *cdtB*, *cdtC*, *virB11*, *pldA*, *cgtB*	13450
CC08	2018	Environment	AM-NAL-CIP-ERI-TET-CHL- LIN	*fexA*, *cfr*, *optrA*, RE-*cmeABC*, *tetO*, *bla*_OXA−61_, *ermB*, *aac(6′)-Ib*, Thr-86-Ile, Ala-2075-Gly	*cadF*, *ciaB*, *racR*, *flaA*, *dnaJ*, *cdtA*, *cdtB*, *cdtC*, *virB11*, *pldA*,	13450

## Data Availability

The data used to support the findings of this study are available from the corresponding author upon reasonable request.
